# DNA-binding miniproteins based on zinc fingers. Assessment of the interaction using nanopores†
†Electronic supplementary information (ESI) available. See DOI: 10.1039/c7sc05441f


**DOI:** 10.1039/c7sc05441f

**Published:** 2018-04-10

**Authors:** Jéssica Rodríguez, Soraya Learte-Aymamí, Jesús Mosquera, Garbiñe Celaya, David Rodríguez-Larrea, M. Eugenio Vázquez, José L. Mascareñas

**Affiliations:** a Centro Singular de Investigación en Química Biolóxica e Materiais Moleculares (CIQUS) , Departamento de Química Orgánica , Universidade de Santiago de Compostela , 15782 Santiago de Compostela , Spain . Email: joseluis.mascarenas@usc.es; b Biofisika Institute (CSIC, UPV/EHU) , Department of Biochemistry and Molecular Biology (UPV/EHU) , Leioa 48940 , Spain

## Abstract

Obtaining artificial proteins that mimic the DNA binding properties of natural transcription factors could open new ways of manipulating gene expression at will. In this context it is particularly interesting to develop simple synthetic systems. Inspired by the modularity of natural transcription factors, we have designed synthetic miniproteins that combine the zinc finger module of the transcription factor GAGA and AT-hook peptide domains. These constructs are capable of binding to composite DNA sequences of up to 14 base pairs with high affinity and good selectivity. In particular, we have synthesized three different chimeras and characterized their DNA binding properties by electrophoresis and fluorescence anisotropy. We have also used, for the first time in the study of peptide-based DNA binders, nanopore force spectroscopy to obtain further data on the DNA interaction.

## Abstract 2

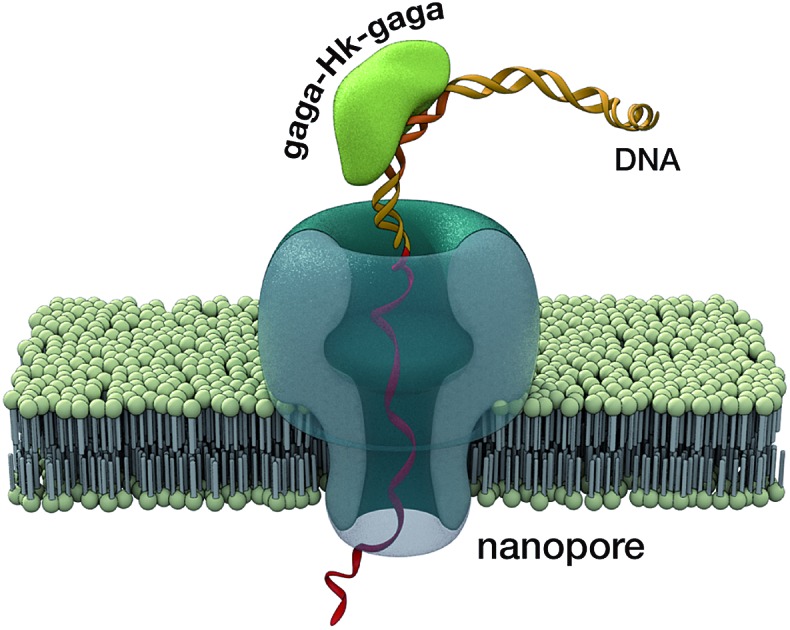
We report a synthetic miniprotein that combines zinc finger modules of the transcription factor GAGA with the AT-hook peptide. This designed chimera binds to extended DNA sites with high affinity and selectivity, as shown by nanopore force spectroscopy.

## 

Zinc fingers (ZFs), the most abundant eukaryotic transcription factors (TFs), are involved in the regulation of the expression of multiple genes.^1^ Cys_2_–His_2_ ZFs are composed of small peptide domains, of about 30 amino acids, which fold into simple ββα-motifs stabilized by chelation of Zn(ii) ions by Cys and His residues. The DNA binding of these proteins typically requires the cooperative interaction of at least three zinc finger domains that wind around the DNA while inserting their recognition α-helices in the major groove, each of them specifically recognizing three base pairs.^2^,^3^ Cys_2_–His_2_ ZFs are a versatile platform for the engineering of genetically encoded transcriptional regulators and gene editing tools, some of which have even reached clinical trials.^3^

Despite the interest in these recombinant constructs, the use of only one class of DNA binding motifs limits the modes of interaction that can be achieved. Therefore, it would be of great value to generate alternative DNA binding agents that can combine different DNA interacting units.^4^,^5^ We have recently demonstrated that, while isolated peptides derived from the GAGA Cys_2_–His_2_ ZF fail to bind DNA, their covalent tethering to minor groove binders such as polypyrroles,^6^ bisbenzamidines^7^ or AT-hook peptide domains^8^ restores their DNA binding. Unfortunately, the synthesis of these conjugates is far from trivial, requiring the use of orthogonal protecting groups and the introduction of elaborate synthetic linkers. Moreover, their non-peptidic nature prevents the future possibility of biological engineering and genetic encoding. These limitations have raised the question of whether it would be possible to assemble analogues of this multipartite DNA binders relying exclusively on natural amino acids and peptide linkers. Herein, we report the synthesis of fully peptidic, ZF-based miniproteins that interact with the DNA with high affinity and excellent selectivity. In contrast to classical ZFs, which only establish contact with the DNA major groove, our designed constructs combine interactions in the major and the minor grooves. We also report for the first time the application of nanopore force spectroscopy to analyze the DNA interaction of this type of artificial peptide DNA binders.^9^

The newly designed chimeras are composed of one AT-hook sequence connected to two Cys_2_–His_2_ replicas of the DNA-binding domain of the GAGA TF (Ser^28^ to Phe^58^ in the reference pdb structure).^10^ Importantly, neither of the components is capable of interacting with their respective DNA sites with appreciable affinity as isolated monomers.5a,^11^ Taking as starting points the experimental structures of the DNA complexes of GAGA,^9^ and the third AT-hook of HMG-I(Y),^12^ we built a hypothetical model for simultaneous interaction of the AT-hook motif inserted into a central AATT minor groove site, flanked by two Cys_2_–His_2_ GAGA fragments bound to adjacent major grooves (see Fig. 1a and the ESI for details†). Inspection of this qualitative model suggested that a Gly_4_ linker between the C-terminal end of the Cys_2_–His_2_ GAGA fragment and the N-terminal arm of the AT-hook might span the required distance. This model also revealed a potentially damaging steric clash involving side chains in the β-hairpin of the second Cys_2_–His_2_ GAGA domain with the C-terminal Lys^40^ of the AT-hook, which was therefore replaced by a glycine (**Hk**^**G**^). In order to maintain the total positive charge of the AT-hook in the final conjugate and favor electrostatic contact with the phosphate backbone, we introduced a lysine residue in the linker connecting the C-terminus of the AT-hook peptide and the N-terminal side of the GAGA fragment (Fig. 1).

**Fig. 1:**
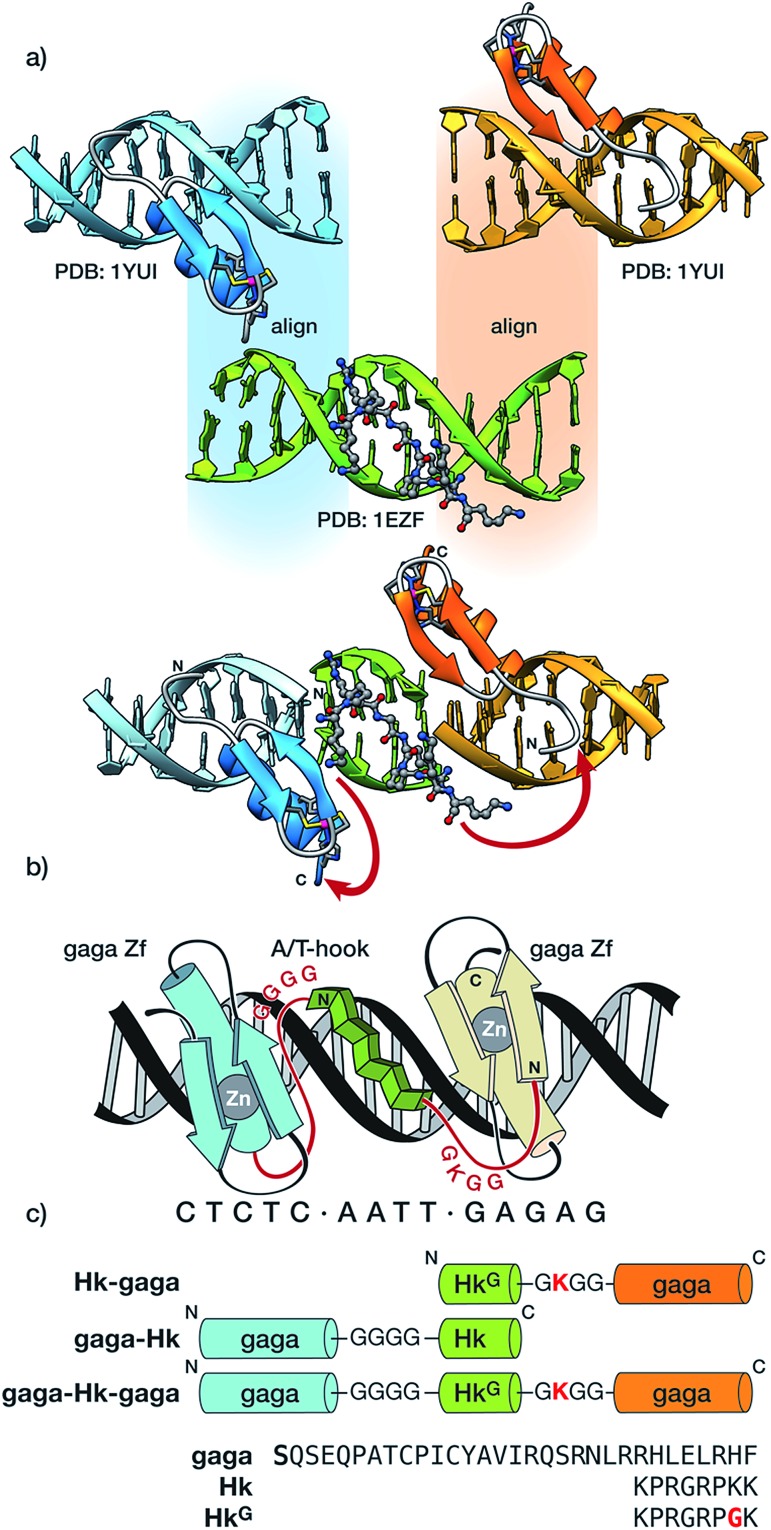
(a) Outline of the structure-guided design consisting of the superposition of the DNA chains of the structures involved in the chimera, followed by cleanup of the overlapping DNA strands and indication of the conjugation scheme between the different DNA binding modules; (b) schematic illustration of the hypothetical tripartite major–minor–major groove recognition by a gaga/AT-hook/gaga chimera. The sequence of the engineered peptide linkers connecting the GAGA DNA binding domains with the central AT-hook anchor is highlighted in red; and (c) schematic representation of the synthesized hybrids and sequences of the modules. Note: molecular modeling considerations suggested that in the case of the C-terminal gaga domains (in orange), it is better to skip the N-terminal Ser residue (indicated in bold in the sequence), in order to direct the linker towards the C-terminus of the AT-hook.

We validated our approach by designing three chimeras, **Hk-gaga**, **gaga-Hk**, and **gaga-Hk-gaga**, which were synthesized in good yields following standard Fmoc/*t*Bu SPPS protocols.^13^ Note that whereas in the first hybrid, **Hk-gaga**, the connection involves the N-terminus of the zinc finger and the C-terminus of the AT-hook, in **gaga-Hk** there is a linkage between the AT-hook N-terminal side and the zinc finger C-terminus. Importantly, synthetic procedures are straightforward and the peptides can be assembled using an automatic synthesizer in just one working day (each peptide), which is an important advantage with respect to previous approaches to conjugates containing non-peptide linkers and binders.^6^,7a

Having at hand the desired bivalent conjugates, we studied their DNA binding properties using non-denaturing electrophoresis mobility shift assays (EMSAs) in polyacrylamide gels.^14^ Thus, a double stranded (ds) oligonucleotide ***AT·GAG*** containing the AT-hook and GAGA binding sites in tandem was mixed with increasing concentrations of the conjugate **gaga-Hk**. The gel showed concentration-dependent slow-migrating bands, which are consistent with the formation of the desired **gaga-Hk**/***AT·GAG*** complex (Fig. 2, panel (a)). Importantly, no new bands were observed when the conjugate **gaga-Hk** was incubated with a dsDNA lacking the GAGA binding site (Fig. 2, panel (c)), demonstrating that the ZF peptide must be bound to its target site for the observation of high-affinity binding. Interestingly, incubation with a control oligonucleotide lacking the A/T-rich site also led to retarded bands, albeit in this case the interaction appears to be weaker (Fig. 2, panel (b) and lanes 2 and 5 in panel (d)). As expected, in the absence of the AT-hook unit, the zinc finger module of GAGA (**gaga**) by itself does not give rise to slow-migrating bands, neither with ***AT·GAG*** nor with ***cg·GAG*** (Fig. 2, panel (d), lanes 3 and 6).

**Fig. 2:**
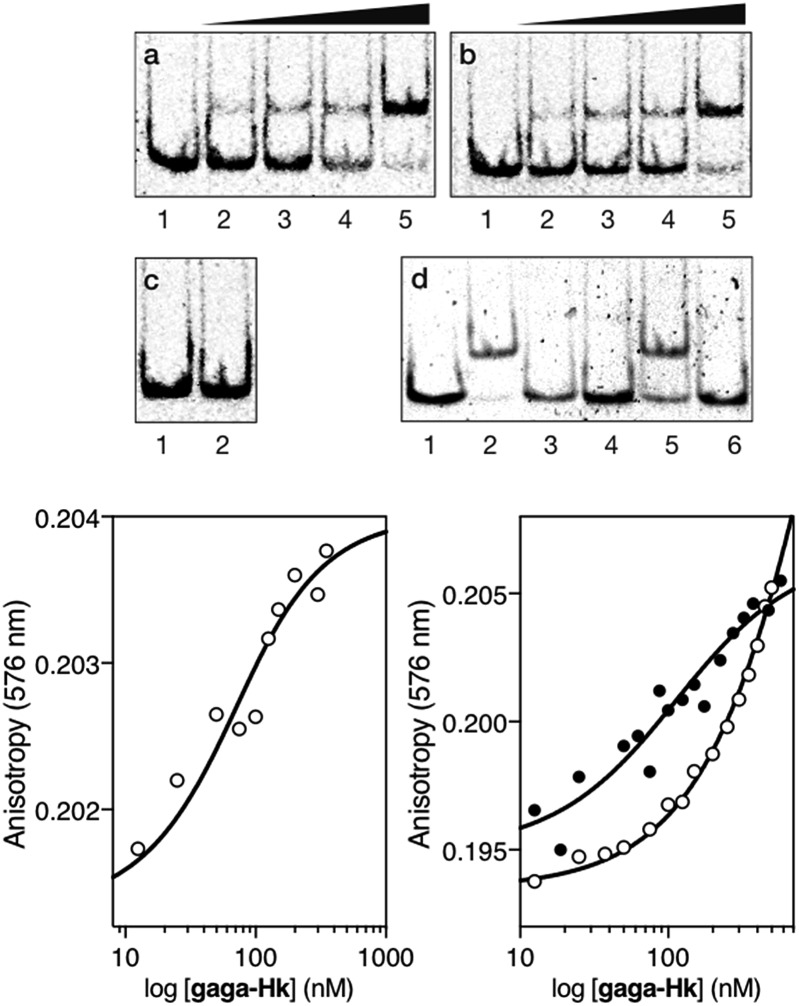
Top: DNA binding of **gaga-Hk** by polyacrylamide gel electrophoresis. Panel (a), lanes 1–5: [**gaga-Hk**] = 0, 300, 500, 700, and 1000 nM with 75 nM ds-oligonucleotide ***AT·GAG***. Panel (b), lanes 1–5: [**gaga-Hk**] = 0, 300, 500, 700, and 1000 nM with 75 nM DNA ***cg·GAG***. Panel (c), lanes 1–2: [**gaga-Hk**] = 700 and 1000 nM with 75 nM DNA ***AT·cgc***. Panel (d), lanes 1–3: 75 nM ***AT·GAG***; lane 2: 1000 nM **gaga-Hk** and lane 3: 1000 nM **gaga**; lanes 4–6: 75 nM ***cg·GAG***; lane 5: 1000 nM **gaga-Hk** and lane 6: 1000 nM **gaga**. Oligonucleotide sequences (only one strand shown, consensus sites underlined): ***AT*·*GAG***: 5′-CGCGTCAT AATT GAGAG CGC-3′; ***AT*·*cgc***: 5′-CGCGTCAT AATT *CGCGA* CGC-3′; ***cg*·*GAG***: 5′-CGCGTCAT *CAGC* GAGAG CGC-3′. Incubations were carried out in 18 mM Tris–HCl buffer (pH 7.5), 90 mM KCl, 1.8 mM MgCl_2_, 0.2 mM TCEP, 9% glycerol, 0.11 mg mL^–1^ BSA, 2.2% NP-40 and 0.02 mM ZnCl_2_. After 30 min at 20 °C, products were resolved by PAGE on a 10% non-denaturing polyacrylamide gel and 0.5× TBE buffer over 40 min at 20 °C, and analyzed by staining with SyBrGold. Bottom left: fluorescence anisotropy titration of a 25 nM solution of TMR-***AT·GAG*** in the presence of competing non-specific calf-thymus DNA (50 μM in base pairs) and with increasing concentrations of **gaga-Hk**. The best fit to a 1 : 1 binding model is also shown. Bottom right: fluorescence anisotropy titration of a 25 nM solution of TMR-***cg·GAG*** in the absence (black circles) and presence of competing non-specific calf-thymus DNA (white circles) (50 μM in base pairs), with increasing concentrations of **gaga-Hk**. Experimental data correspond to the mean of three independent experiments.

Fluorescence anisotropy titrations with a rhodamine (TMR)-labeled dsDNA containing the target consensus sequence (AATT-GAGAG) confirmed that **gaga-Hk** binds with high affinity to its target site, with an apparent *K*_D_ of 58 ± 4 nM in the presence of competing calf thymus DNA (41 ± 7 nM in the absence of calf thymus, see the ESI†) at 20 °C (Fig. 2, bottom left). Importantly, fluorescence anisotropy titrations showed that in the presence of excess competing calf thymus DNA, **gaga-Hk** binds very weakly to the mutated dsDNA lacking the A/T-rich tract (Fig. 2, bottom right, white points) while in the absence of calf thymus the data can be fitted with a *K*_D_ of 100 nM (Fig. 2, bottom right, black points). This result indicates that the retarded band observed in the EMSA with this mutated DNA (Fig. 2b) arises from relatively weak and less specific interactions in which the AT-hook peptide is most probably not inserted in the minor groove, but makes electrostatic contact with the DNA backbone.^15^ Taken together, these results support the formation of a cooperative, bivalent DNA binding complex at specific composite DNA sites of nine base pairs (AATT-GAGAG), in which the GAGA peptide fragment binds in the major groove of its target sequence (GAGAG) while the AT-hook peptide is inserted in the adjacent minor groove (AATT).

The inverted chimera **Hk-gaga** also targets the same composite DNA site. Thus, the addition of increasing amounts of **Hk-gaga** to the dsDNA ***AT·GAG*** under standard conditions led to a new, slow-migrating band (Fig. 3, panel (a)). This new band is consistent with the expected specific peptide–DNA complex. As previously observed for the conjugate **gaga-Hk**, **Hk-gaga** does not elicit new retarded bands when incubated with a non-target sequence lacking the GAGAG site (Fig. 3, panel (b)), and shows residual binding with a control oligonucleotide featuring the GAGAG site but lacking the A/T-rich site (Fig. 3, panel (c)). Therefore, the inverse arrangement of DNA binding moieties allowed an excellent selectivity. Using fluorescence anisotropy, we calculated an apparent *K*_D_ for its target site of 92 ± 11 nM at 20 °C, in the presence of competing calf thymus DNA (44 ± 6 nM in the absence of calf thymus; Fig. 3, bottom left, and ESI†). As with **gaga-Hk**, in the presence of calf thymus, the interaction of **Hk-gaga** with the DNA featuring the A/T-hook mutated site is very weak (Fig. 3, bottom right). Taken together, these results confirm the formation of the expected bivalent complex at the specific composite DNA site of nine base pairs (complex **Hk-gaga**/***AT·GAG***).

**Fig. 3:**
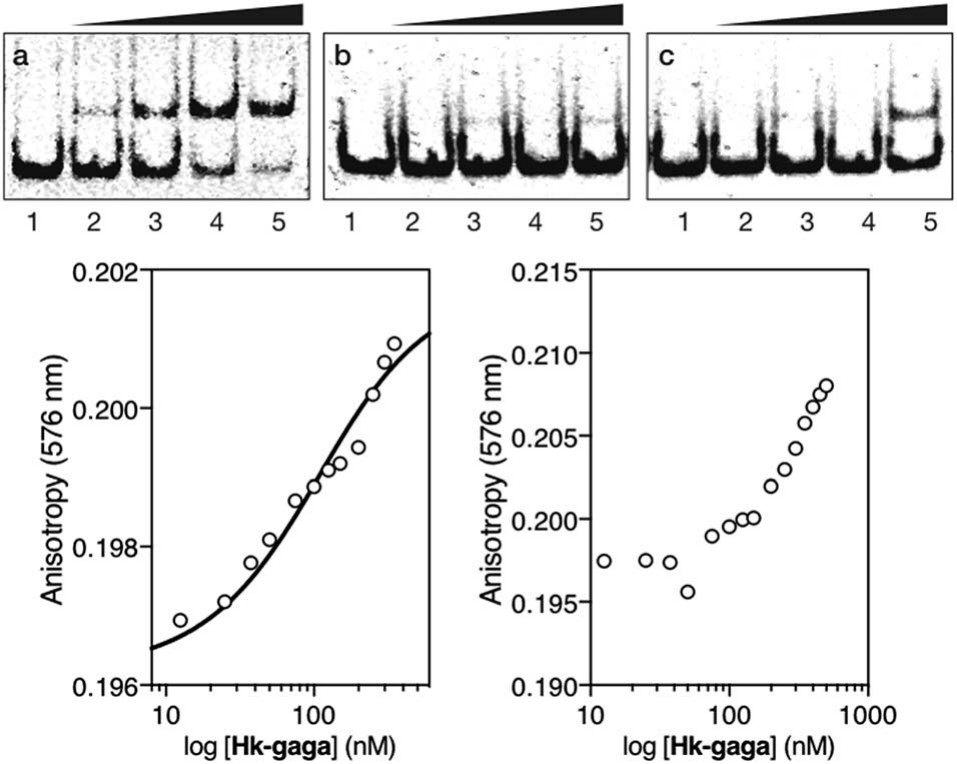
Top: EMSA results for **Hk-gaga**. Panel (a), lanes 1–5: [**Hk-gaga**] = 0, 300, 500, 700, and 1000 nM with 75 nM DNA ***AT·GAG***. Panel (b), lanes 1–5: [**Hk-gaga**] = 0, 300, 500, 700, and 1000 nM with 75 nM ***AT·cgc***. Panel (c), lanes 1–5: [**Hk-gaga**] hr = 0, 300, 500, 700, and 1000 nM with 75 nM ***cg·GAG***. Experimental conditions are as indicated in the caption of Fig. 2. Bottom left: fluorescence anisotropy titration of a 25 nM solution of TMR-***AT·GAG*** in the presence of competing non-specific calf-thymus DNA (50 μM in base pairs) and with increasing concentrations of **Hk-gaga**. The best fit to a 1 : 1 binding model is also shown. Bottom right: fluorescence anisotropy titration of a 25 nM solution of TMR-***cg·GAG*** in the presence of competing non-specific calf-thymus DNA (50 μM in base pairs) and with increasing concentrations of **Hk-gaga**. Experimental data correspond to the mean of three independent titrations. Note the different concentration scale that reflects the very different binding properties with the two oligos.

We then moved to the more challenging ternary “major–minor–major” groove interaction. In this case, the AT-hook plays the role of a central minor groove anchor that delivers the two GAGA DNA binding domains to the adjacent major groove sites. Gratifyingly, the addition of increasing concentrations of the ternary chimera **gaga-Hk-gaga** to a dsDNA containing the palindromic target composite binding site (CTC·AT·GAG) under standard conditions, produced a new, slower migrating band in the EMSA (Fig. 4, panel (a), lanes 1–6), consistent with the formation of the expected specific ternary miniprotein/DNA complex. With the mutated dsDNA ***cat·AT·GAG***, which lacks the first GAGAG site, the gel shows a faint, slower-migrating band, that might correspond to a lower-affinity peptide/DNA complex involving a specific bivalent interaction with the target AATT-GAGAG sequence (Fig. 4, panel (b), lanes 1–2). Importantly, the synthetic miniprotein does not elicit retarded bands when incubated with a non-target sequence lacking both GAGA binding sites, ***cat·AT·cgc*** (Fig. 4, panel (c), lanes 1–5). Again, a control oligonucleotide lacking the A/T-rich site, ***CTC·gc·GAG***, showed only residual binding (Fig. 4, panel (c), lanes 6–10). This result highlights the important role of the interaction between the AT-hook moiety of the conjugate with its target site, for obtaining high affinity complexes.

**Fig. 4:**
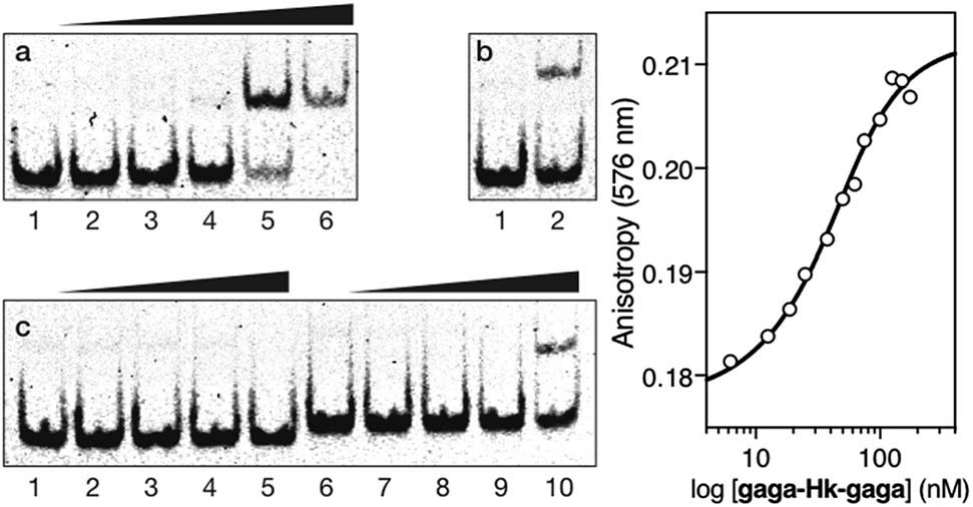
Left: EMSA results for **gaga-Hk-gaga**. Panel (a), lanes 1–6: [**gaga-Hk-gaga**] = 0, 300, 500, 700, 1000, and 1500 nM with 75 nM ***CTC·AT·GAG***. Panel (b), lanes 1–2: [**gaga-Hk-gaga**] = 0 and 1000 nM with 75 nM ***cat·AT·GAG***. Panel (c), lanes 1–5: [**gaga-Hk-gaga**] = 0, 300, 500, 700, and 1000 nM with 75 nM ***cat·AT·cgc***. Panel (c), lanes 6–10: [**gaga-Hk-gaga**] = 0, 300, 500, 700, and 1000 nM with 75 nM ***CTC·gc·GAG***. Oligonucleotide sequences (only one strand shown): ***CTC·AT·GAG***: 5′-GAG CTCTC *AATT* GAGAG CGCG-3′; ***cat·AT·GAG***: 5′-CGC GTCAT *AATT* GAGAG CGC-3′; ***cat·AT·cgc***: 5′-CGC GTCAT *AATT* CGCGA CGC-3′; ***CTC·gc·GAG***: 5′-GGTT CTCTC *GACC* GAGAG TTGG-3′. Experimental conditions are as indicated in the caption of Fig. 2. Right: Fluorescence anisotropy titration of a 25 nM solution of TMR-***CTC·AT·GAG*** with increasing concentrations of **gaga-Hk-gaga** and best fit to a 1 : 1 binding model. Data are the mean of three titrations.

**Fig. 5:**
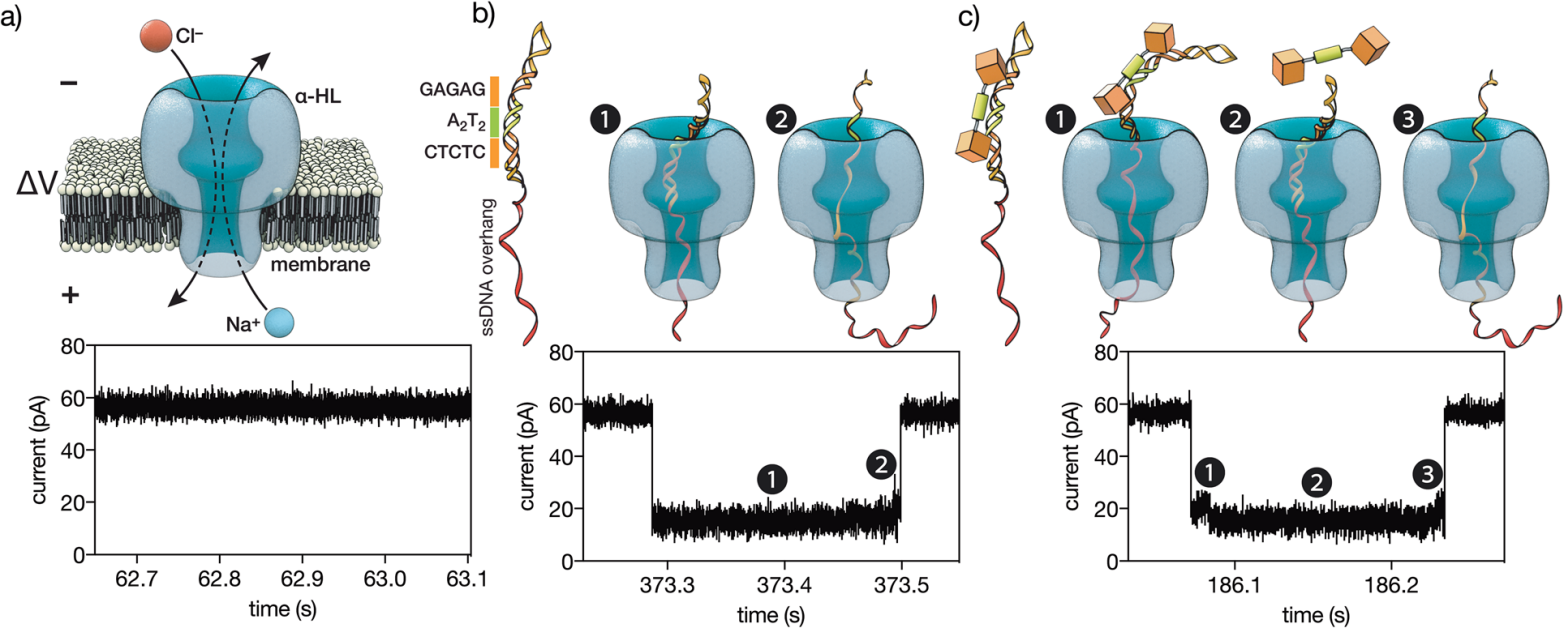
(a) A single α-HL pore inserted in a lipid membrane allows the flow of ions in response to an applied voltage. The ionic current of the open pore is shown below. (b) A double stranded DNA containing the target CTCTC-A_2_T_2_-GAGAG binding site inserted in a hairpin followed by a ssDNA overhang (see the ESI† for the full sequence). This DNA is driven to the pore by the electric field. The threading, unzipping and translocation of the DNA causes the characteristic signal shown below. (c) When the trivalent peptide is bound to the DNA there is an additional step with the DNA–protein complex on top of the pore, which causes a new high-conductance level (c1). Once the protein is detached, the reaction proceeds as with free DNA (c2-3). Below is the ionic current signal when a protein–DNA complex is analyzed.

Fluorescence anisotropy titrations using a TMR-labeled dsDNA (TMR-***CTC·AT·GAG***) confirmed the high affinity binding of the trivalent peptide chimera with the DNA (apparent *K*_D_ = 35 ± 4 nM at 20 °C, Fig. 4, right). We could not calculate a reliable *K*_D_ in the presence of excess calf-thymus DNA because of the formation of aggregates. Anyhow, these results support the formation of a trivalent DNA complex at the specific composite consensus DNA site of 14 base pairs. The lack of large enhancement in the binding affinity of the ternary chimera (**gaga-Hk-gaga**) *versus* the bivalent systems (**Hk-gaga** and **gaga-HK**) is likely due to the use of a suboptimal linker that does not allow full energetic advantage of the simultaneous interaction of the three binding modules to be taken.

We next analyzed the interaction of the trivalent chimera with DNA using nanopores.^16^ This single molecule method has shown utility for the determination of thermodynamic and kinetic parameters in the formation of protein–DNA complexes,^17^ and to our knowledge, up to now it had not been used to characterize the DNA recognition of synthetic peptide binders.9*c* Briefly, it works by stochastically examining DNA states in the presence of a given amount of the peptide binder, as described in Fig. 5. Typically, by analyzing up to a hundred DNA molecules, the fraction of complexes can be determined (Fig. 6, left), and the *K*_D_ deduced (Fig. 6, right). When analyzing the interaction of the trivalent chimera with its target ternary binding site 
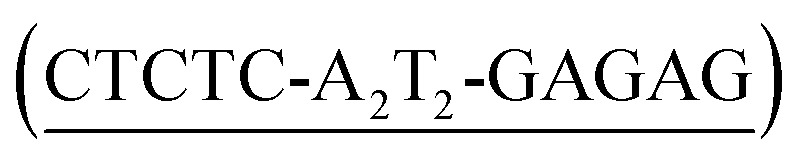
 we calculated a *K*_D_ of 120 ± 10 nM. This result is in reasonable agreement with the *K*_D_ obtained by fluorescence anisotropy, considering the differences in the assay conditions and in the characteristics of each technique. As expected, mutation of the first GAGAG site (*GTCAT*-A_2_T_2_-GAGAG) or of the second GAGAG site (CTCTC-A_2_T_2_-*CTGGG*) led to weaker affinities (*K*_D_ of 193 ± 66 nM and 269 ± 39 nM, respectively), in agreement with the trend in binding affinity observed in the EMSA experiments. Interestingly, the nanopore technique allows the time required for a protein to detach from its DNA complex to be measured (dwell time in level 1 in Fig. 4c). This information is related to the kinetics in the presence of an applied force.9c,^17^ In order to compare the data obtained on the three different DNAs we fitted each dwell time distribution to a single exponential distribution (ESI Fig. S6†). The values obtained from the fit should be taken cautiously because between 10 and 20% of the molecules did not fit the single exponential distribution. Overall, we observed that higher voltages induced faster dissociation, likely because of the increased force applied to detach the protein under those conditions (Fig. S7†). The effect of the applied force was larger when the protein was bound to the DNA with the consensus tripartite site (CTCTC-A_2_T_2_-GAGAG). Within the voltage range studied (from +90 to +120 mV), the slower dissociation was also observed for this DNA (apparent *k*_off,110 mV_ = 90[100 – 79] s^–1^; in brackets the 95% confidence interval, CI; *n* = 284; Fig. S6†). For the DNAs with one mutated site the dissociation was faster (for DNA with *GTCAT*-A_2_T_2_-GAGAG, apparent *k*_off,110 mV_ = 165[197 – 132] s^–1^ ([95% CI], *n* = 100) and for that with CTCTC-A_2_T_2_-*CTGGG*, apparent *k*_off,110 mV_ = 253[298 – 208] s^–1^ ([95% CI], *n* = 121)).

**Fig. 6:**
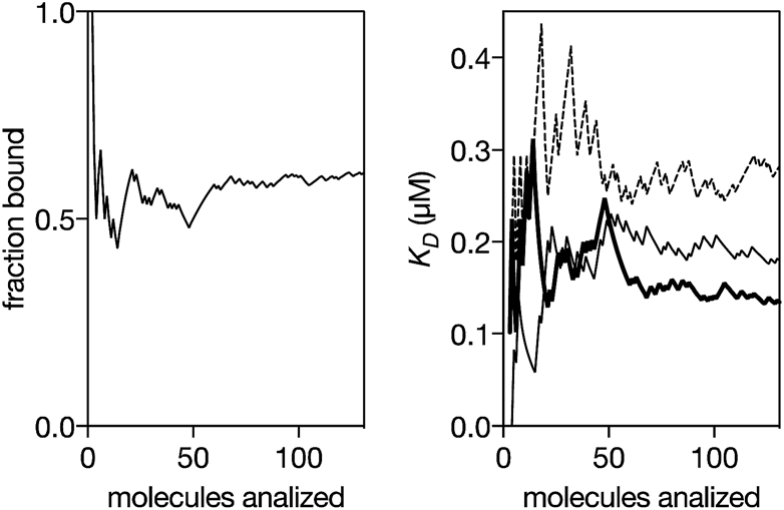
Left: Representative experiment where DNA molecules are stochastically analyzed by the pore. The fraction of peptide–DNA complexes in a mixture of 0.29 μM peptide and 0.13 μM DNA is calculated counting the number of molecules that produced the signal shown in Fig. 5c divided by the total number of molecules that were analyzed (Fig. 5b and c). Using a reversible one-to-one binding model, *K*_D_ = [free DNA] × [free protein]/[complex], with [DNA] = total concentration of DNA, [Prot] = total concentration of protein and *f*_b_ = fraction of DNA molecules observed in the bound state, we estimated an apparent *K*_D_ = [DNA] × (1 – *f*_b_) × ([Prot]–[DNA] × *f*_b_)/([DNA] × *f*_b_) (black thick line in the right panel). Right: Representative experiments for the *K*_D_ estimation for a DNA with the target ternary binding site (thick line, 0.29 μM peptide and 0.13 μM DNA), for a DNA with the 1^st^ GAGAG site mutated (thin line, 0.52 μM peptide and 0.13 μM DNA) and for a DNA with the second GAGAG site mutated (dashed line, 0.19 μM peptide and 0.13 μM DNA). The experiments were carried out in 100 mM NaCl, 0.02 mM ZnCl_2_, 20 mM Tris–HCl pH 7.5, at 22 °C.

## Conclusions

In summary, we have devised a new type of fully-peptidic DNA binder with a new artificial DNA binding motif. Bivalent and trivalent constructs can be prepared in a straightforward manner owing to their peptidic constitution, and display excellent DNA recognition properties in terms of affinity and selectivity. In addition, we have shown that nanopore technologies allow biophysical information to be obtained, in particular kinetic information that complements that obtained using more standard ensemble techniques. We predict that the proteinogen nature of these artificial DNA binders might allow further designs, and provide for the development of genetic tools other than those based on polydactyl zinc fingers.

## Conflicts of interest

There are no conflicts to declare.

## Supplementary Material

Supplementary information
