# Supplementary material for: DNA-binding miniproteins based on zinc fingers. Assessment of the interaction using nanopores
Source: Chem Sci. 2018 Apr 10;9(17):4118–23. doi: 10.1039/c7sc05441f (PMC5941273; doi:10.1039/c7sc05441f)
Supplement: Supplementary file 1 [file SC-009-C7SC05441F-s001.pdf]

Electronic Supplementary Material (ESI) for Chemical Science.
This journal is © The Royal Society of Chemistry 2018

DNA-binding miniproteins based on Zinc Fingers. Assessment of the interaction using nanopores

Jéssica Rodríguez,[a] Soraya Learte-Aymamí,[a] Jesús Mosquera,[a] Garbiñe Celaya,[b] David Rodríguez-Larrea,[b] M. Eugenio Vázquez,*,[a] and José L. Mascareñas*,[a]

[a] Centro Singular de Investigación en Química Biolóxica e Materiais Moleculares (CIQUS), Departamento de Química Orgánica. Universidade de Santiago de Compostela, 15782 Santiago de Compostela, Spain.
[b] Biofisika Institute (CSIC, UPV/EHU) and Department of Biochemistry and Molecular Biology (UPV/EHU), Leioa 48940, Spain

1. Abbreviations S2
2. General peptide synthesis procedures S2
3. Superpositions S5
4. Oligonucleotide sequences S5
5. EMSA experiments S6
6. Fluorescence Anisotropy S6
7. Fluorescence anisotropy curve fitting S7
8. Single-molecule nanopore experiments S8

1. Abbreviations

HBTU: 2-[(1H-benzotriazol-1-yl)-1,1,3,3-tetramethyluronium hexafluorophosphate

TMR: tetramethylrhodamine dye

TFA: trifluoroacetic acid

TIS: triisopropylsilane

TMR =

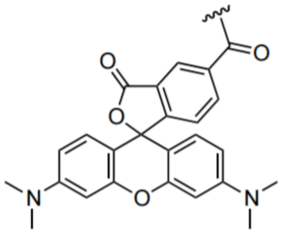

2. General peptide synthesis procedures

All peptide synthesis reagents and amino acid derivatives were purchased from GL Biochem (Shanghai) and Novabiochem; amino acids were purchased as protected Fmoc amino acids with the standard side chain protecting scheme: Fmoc-Ala-OH, Fmoc-Leu-OH, Fmoc-Lys(Boc)-OH, Fmoc-Ser(*t*-Bu)-OH, Fmoc-Glu(O*t*-Bu)-OH, Fmoc-Trp(Boc)-OH, Fmoc-Asn(Trt)-OH, Fmoc-Ile-OH, Fmoc-Thr(*t*-Bu)-OH, Fmoc-Arg(Pbf)-OH, Fmoc-His(Trt)-OH and Fmoc-Asp(O*t*-Bu)-OH. All other chemicals were purchased from *Aldrich* or *Fluka*. All solvents were dry and synthesis grade, unless specifically noted.

Peptides were synthesized using an automatic peptide synthesizer from *Protein Tecnologies PS3 PeptideSynthesizer*. Peptide synthesis was performed using standard Fmoc solid-phase method on a PAL–PEG–PS resin (0.19 mmol/g) using HBTU/HOBt (4 equiv) as coupling agent, DIEA as base (6 equiv) and DMF as solvent. The deprotection of the temporal Fmoc protecting group was performed by treating the resin with 20% piperidine in DMF solution for 20 min.

The cleavage/deprotection step was performed by treatment of the resin-bound peptide for 1.5–2h with the following cleavage cocktail: 940 µL TFA, 25 µL EDT, 25 µL H_2O and 10 µL TIS (1 mL of cocktail / 40 mg resin).

The crude products were purified by RP−HPLC, 4 mL/min, gradient 10 to 50% B over 40 min. (A: H_2O 0.1% TFA, B: CH_3CN 0.1% TFA) and identified as the desired peptides.

High-Performance Liquid Chromatography (HPLC) was performed using an *Agilent 1100* series Liquid Chromatograph Mass Spectrometer system. Analytical HPLC was carried out using a *Eclipse XDB-C18 analytical column (4.6 x 150 mm, 5 µm)*, 1 mL/min, gradient 5 to 75% B over 30 min. Purification of the peptides was performed on a semipreparative *Phenomenex Luna–C18* (250 × 10 mm) reverse-phase column.

Electrospray Ionization Mass Spectrometry (ESI/MS) was performed with an *Agilent 1100* Series LC/MSD VL G1956A model in positive scan mode.

gaga: H-**SQSEQPATCP ICYAVIRQSR NLRRHLELRH F**-NH_2

Was isolated with an approx. yield of 21%.

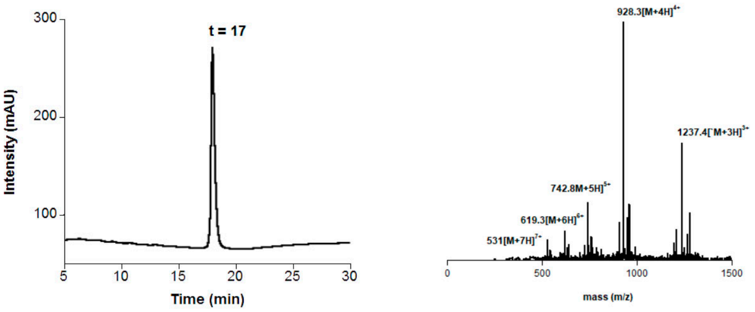

Figure S1. Left) HPLC chromatogram of purified peptide. Gradient 5 to 75% B over 30 min. Right) Mass spectrum of the purified peptide.

EM–ESI$^+$ (m/z): Calcd. for $C_{159}H_{258}N_{54}O_{45}S_2$: 3710. Found: 1237.4 $[M+4H]^3$; 928.3 $[M+4H]^{4+}$; 742.8 $[M+5H]^{5+}$; 619.3$[M+6H]^{6+}$; 531 $[M+7H]^{7+}$.

gaga-Hk: H-**SQSEQPATCP ICYAVIRQSR NLRRHLELRH FGGGGKPRGR PKK**-NH_2

Was isolated with an approx. yield of 19%.

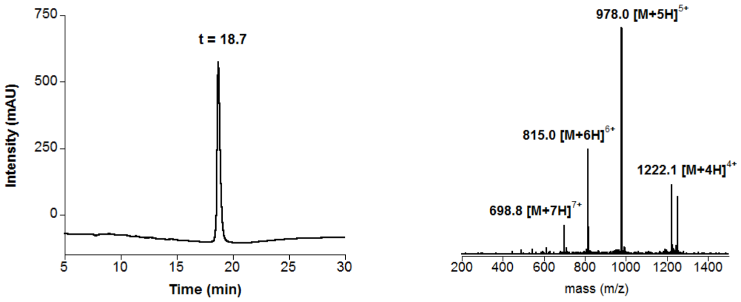

Figure S2. Left) HPLC chromatogram of purified peptide. Gradient 5 to 75% B over 30 min. Right) Mass spectrum of the purified peptide.

EM–ESI$^+$ (m/z): Calcd. for $C_{209}H_{347}N_{75}O_{57}S_2$: 4883.6. Found: 1222.1 $[M+4H]^{4+}$; 978.0 $[M+5H]^{5+}$; 815.0 $[M+6H]^{6+}$; 698.8 $[M+7H]^{7+}$.

Hk-gaga: H-**KPRGRPGKGK GGSQSEQPAT CPICYAVIRQ SRNLRRHLEL RHF**-$CONH_2$

Was isolated with an approx. yield of 17%.

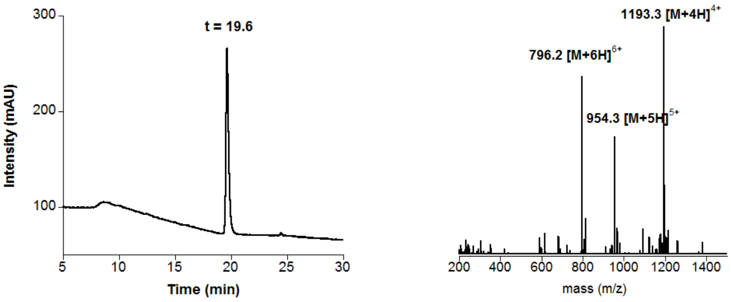

Figure S3. Left) HPLC chromatogram of purified peptide. Gradient 5 to 75% B over 30 min. Right) Mass spectrum of the purified peptide.

EM–ESI$^+$ (m/z): Calcd. for $C_{206}H_{342}N_{72}O_{55}S_2$: 4768.6. Found: 1193.3 $[M+4H]^{4+}$; 954.3 $[M+5H]^{5+}$; 796.2 $[M+6H]^{6+}$.

gaga-Hk-gaga: H-**SQSEQPATCP ICYAVIRQSR NLRRHLELRH FGGGGKPRGR PGKGKGGSQS EQPATCPICY AVIRQSRNLR RHLELRHF**-NH_2

Was isolated with an approx. yield of 10%.

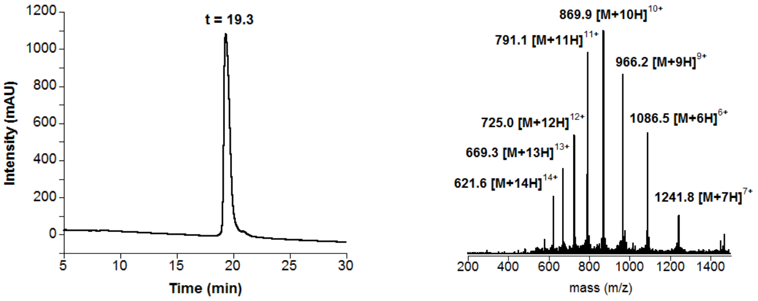

Figure S4. Left) HPLC chromatogram of purified peptide. Gradient 5 to 75% B over 30 min. Right) Mass spectrum of the purified peptide.

EM–ESI$^+$ (m/z): Calcd. for $C_{373}H_{610}N_{130}O_{103}S_4$: 8686.5. Found: 1241.8 $[M+7H]^{7+}$; 1086.5 $[M+8H]^{8+}$; 966.2 $[M+9H]^{9+}$; 869.9 $[M+10H]^{10+}$; 791.1 $[M+11H]^{11+}$; 725.0 $[M+12H]^{12+}$; 669.3 $[M+13H]^{13+}$; 621.6 $[M+14H]^{14+}$.

3. Superpositions

This was carried out with MacPymol using the references PDB ID: 3UXW for AT-Hook and 1YUI for GAGA.

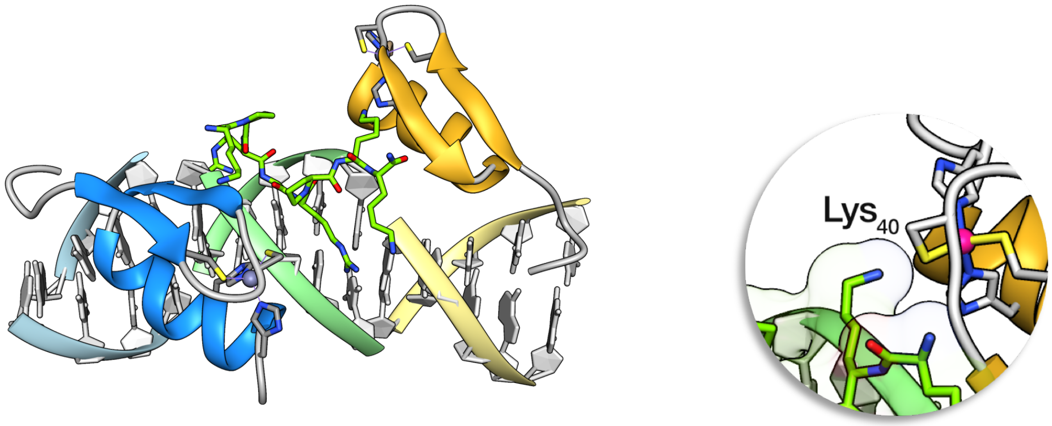

Figure S5. Left) Model of the simultaneous interaction of the **AT-Hook** flanked by two **GAGA** fragments (blue and orange). Right) Zoom of the model showing the damaging steric clash between Lys^{40} of the AT-hook and the beta-hairpin of the second GAGA fragment (see main manuscript for the influence of this fact in the designed peptides)

4. Oligonucleotide sequences

Double stranded (only one strand is shown) oligonucleotides used for EMSA experiments with peptides **gaga-Hk**, **Hk-gaga** and **gaga-Hk-gaga** were supplied by *Thermo Fischer* and their sequences were:

AT•GAG* or *cat•AT•GAG	5´-CGCGTCAT*AATT*GAGAGCGC-3´
AT•cgc* or *cat•AT•cgc	5´-CGCGTCAT*AATT*CGCGACGC-3´
cg•GAG	5´-CGCGTCAT*CAGC*GAGAGCGC-3´
CTC•AT•GAG	5´-GAGCTCTC*AATT*GAGAGCGCG-3´
CTC•gc•GAG	5´-GGTTCTCTC*GACC*GAGAGTTGG-3´

Oligonucleotides used for nanopore experiments were supplied by *Thermo Fischer* and their sequences were:

DNA with the tripartite binding site: 5´-CCCCCCCCCCCCCCCCCCCCAAAAAAAAAAAGAG CTCTC*AATT*GAGAGCGCGCACACACGCGCTCTC*AATT*GAGAGCTC-3´

DNA mutated in the 1st GAGAG site: 5´CCCCCCCCCCCCCCCCCCCCAAAAAAAAAAAGAG GTCAT*AATT*GAGAGCGCGCACACACGCGCTCTC*AATT*ATGACCTC 3´

DNA mutated in the 2nd GAGAG site: 5´CCCCCCCCCCCCCCCCCCCCAAAAAAAAAAAGAG CTCTC*AATT*CTGCCCGCGCACACACGCGGGCAG*AATT*GAGAGCTC 3´

5. EMSA experiments

EMSAs were performed with a BioRad Mini Protean gel system, powered by an electrophoresis power supplies Power Pac Basic model, maximum power 150 V, frequency 50-60 Hz at 140 V (constant V). Binding reactions were performed over 30 min in 18 mM Tris-HCl buffer (pH 7.5), 90 mM KCl, 1.8 mM $MgCl_2$, 0.2 mM TCEP, 9% glycerol, 0.11 mg/mL BSA, 2.2% NP-40 and 0.02 mM of $ZnCl_2$. In the experiments we used 75 nM of the ds−DNAs and a total incubation volume of 20 µL. After incubation for 30 min products were resolved by PAGE using a 10% non-denaturing polyacrylamide gel and 0.5× TBE buffer for 40 min at 20 ºC, and analyzed by staining with SyBrGold (Molecular Probes: 5 µL in 50 mL of 1× TBE) for 10 min and visualized by fluorescence.

5× TBE buffer: 0.445M Tris, 0.445 M Boric acid.

6. Fluorescence Anisotropy

Measurements were made with a Jobin-Yvon Fluoromax-3, (DataMax 2.20) coupled to a Wavelength Electronics LFI−3751 temperature controller, using the following settings: integration time: 2.0 s; excitation slit width: 5.0 nm; emission slit width: 20.0 nm; excitation wavelength 559 nm; emission wavelength 585 nm.

5 µL of a 5 µM water solution of selected **TMR-oligo** (TMR-5´-oligonucleotide-3) were added to 995 µL of Tris-HCl buffer 20 mM pH 7.5, 100 mM NaCl, 0.02 mM of $ZnCl_2$, and calf thymus DNA (when present, 50 µM in base pairs) and the anisotropy was measured at 559 nm, at 20 ºC. Aliquots of a stock solution in water of the corresponding peptide (12.5 µM) were successively added to this solution, and the anisotropic value was recorded after each addition.

7. Fluorescence anisotropy curve fitting

Experimental data were fitted with the *DynaFit 4.0* software, which performs a numerical treatment of the system.[1] The program is available free of charge for academia at http://www.biokin.com/dynafit/.

Dynafit requires plain text files called scripts that contains information about the chemical model underlying the experimental data, the values of model parameters, such as starting concentrations of reactants, as well as information about location of the files. A typical script used in the analysis titrations is included below. The file has been commented to indicate the purpose of the keywords and sections.

```
[task]                     ;semicolons indicate comments not read by the
 task = fit                ;the DynaFit program
 data = equilibria         ;nature of the calculation to be performed

[mechanism]                        ;Free-form 1:1 binding model with Kd
 R + L <==> RL : Kd dissoc         ;to be calculated as dissociation constant

[constants]                ;Initial Kd value for iteration
 Kd = 1.0 ?                ;the "?" indicates that this will be optimized

[concentrations]
 R = 2.0                   ;Fixed conc. of the DNA during the peptide titration

[responses]                ;contribution to the spectroscopic signal of each
 R = 0.1 ?                 ;of the different components of the equilibrium
 RL = 1.5 ?                ;these will be optimized ("?" after the values).

[data]                     ;location of files and information about the data
 variable  L               ;the species that changes conc. during the titration
 offset auto ?
 directory ./exp/EVS/KIT   ;file path (relative to DynaFit program location)
 extension txt
 file f1                   ;name of the experimental data file

[output]
 directory ./exp/EVS/KIT/out ;path indicating location of DynaFit output files

 [settings]                ;cosmetic settings that control DynaFit graphics
 {Output}                  ;fits were exported & finally plotted with
 XAxisUnit = uM            ;GraphPad Prism 7.0c. GraphPad Software,
 BlackBackground = n       ;La Jolla California, www.graphpad.com
 XAxisLabel = [peptide]
 YAxisLabel = anisotropy
 WriteTXT = y
```

[1] a) P. Kuzmič, *Methods in Enzymology*, 2009, **467**, 247–280; b) P. Kuzmic, *Anal. Biochem.,* 1996, **237**, 260–273.

8. Single-molecule nanopore experiments

Single-molecule measurements were performed in planar lipid bilayers made of 1,2-diphytanoyl-sn-glycero-3-phosphocholine (Avanti Polar Lipids) generated with the Montal and Mueller method across an aperture in a Teflon film (0.025 mm thick, Goodfellow) that separated the two compartments of a chamber (*cis* and *trans*). The measurements were recorded at 20 ºC in asymmetric conditions. The *cis* compartment (connected to the ground electrode and where samples were added) was filled with 20 mM Tris-HCl pH 7.5, 100 mM NaCl, 0.02 mM of $ZnCl_2$ and the *trans* compartment with 5 mM $MgCl_2$, 2 M KCl, 10 mM HEPES pH 7.2 (to increase the ionic current signal). 0.5 µL of previously heptamerized α-hemolysisn (αHL) was added to the *cis* compartment and following the first insertion the chamber was manually perfused to remove remaining αHL. Ionic currents through the αHL pore were measured using previously balanced Ag/AgCl electrodes with agar bridges in 3 M KCl. The electrodes were connected to an amplifier (Axopatch 200B, Molecular Devices), the signal was filtered at 5 kHz and recorded at 20 kHz with a digitizer (Digidata 1440A, Molecular Devices). Data was obtained in a range of DNA concentrations (0.12-0.15 µM) and peptide concentrations (0.038-0.5 µM) at different voltages (from +90 mV to 120 mV). Raw data was first analysed with pClamp software (Molecular Devices) to measure the dwell time in the protein-DNA state (level 1 in Fig. 5c). The fraction of protein bound DNA molecules was estimated dividing the number of signals with an initial step as in Fig 5c by the total number of signals corresponding to DNA molecules (Fig 5b + Fig 5c). All dwell times in the protein-DNA state were used to make histograms as shown in Figure S6 (rows 2, 4 and 6). These histograms were fitted using the Curve Fitting routine in Igor Pro (Wavemetrics) and the equation $y = A*exp(-\lambda*x)$ with A = amplitude, λ= rate parameter and x= dwell time. Alternatively the natural logarithm of the dwell time in the protein-DNA state was used to make the histograms as shown in Figure S6 (rows 1, 3 and 5). These histograms were fitted using the equation $y = A*\lambda*exp(x-\lambda*exp(x))$ with A = area; λ= rate parameter; x= ln(dwell time).

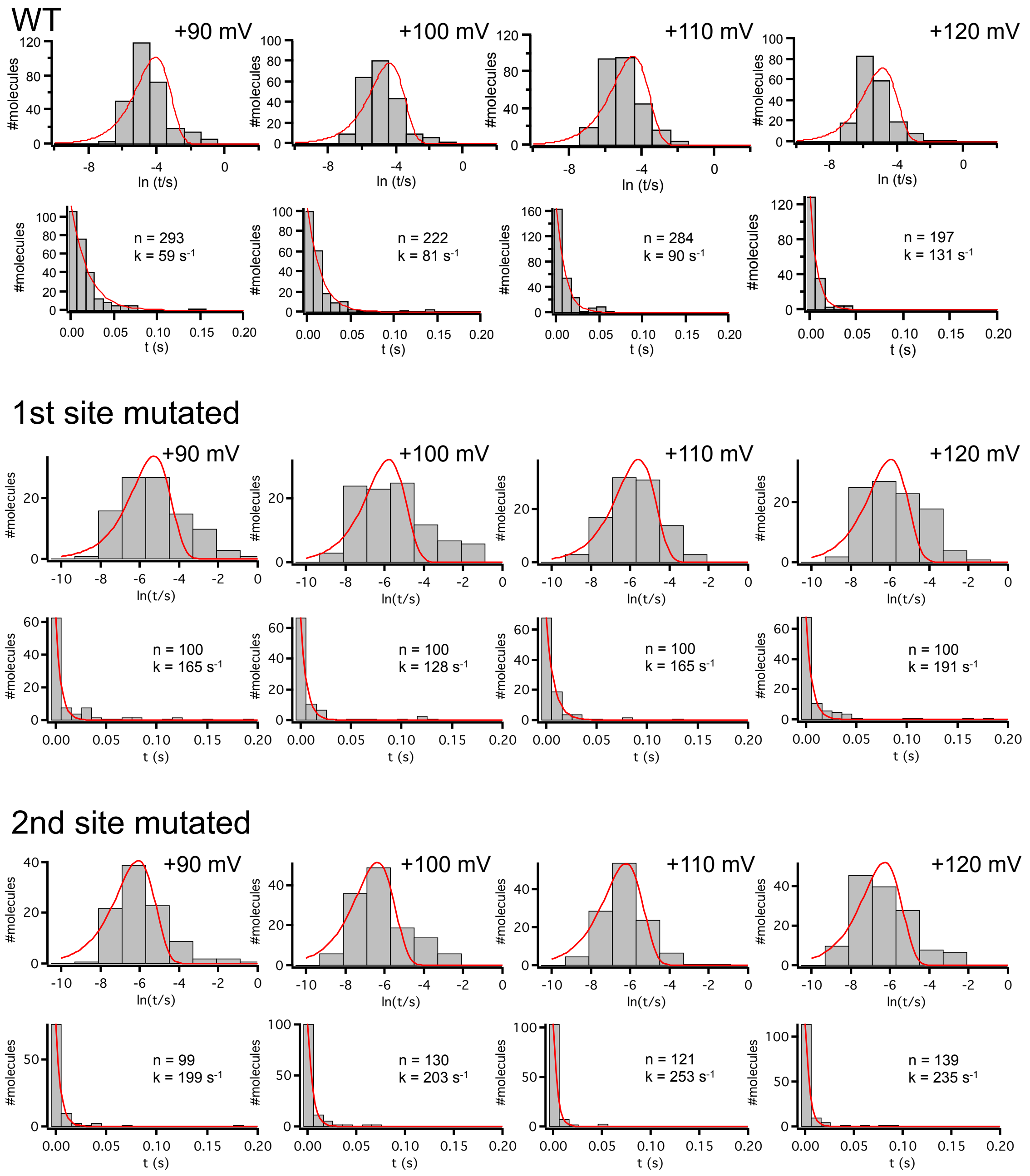

Figure S6. Kinetics (k_{off}) of detachment for the trivalent peptide at different voltages for a DNA that contains the copnsensus tripartite binding site (WT, rows 1 and 2); for a DNA with the 1st GAGAG binding site mutated (rows 3 and 4); and for the DNA with the 2nd GAGAG binding site mutated (rows 5 and 6). The data are displayed both in linear and in logarithmic scale. In red the fit to a single exponential distribution.

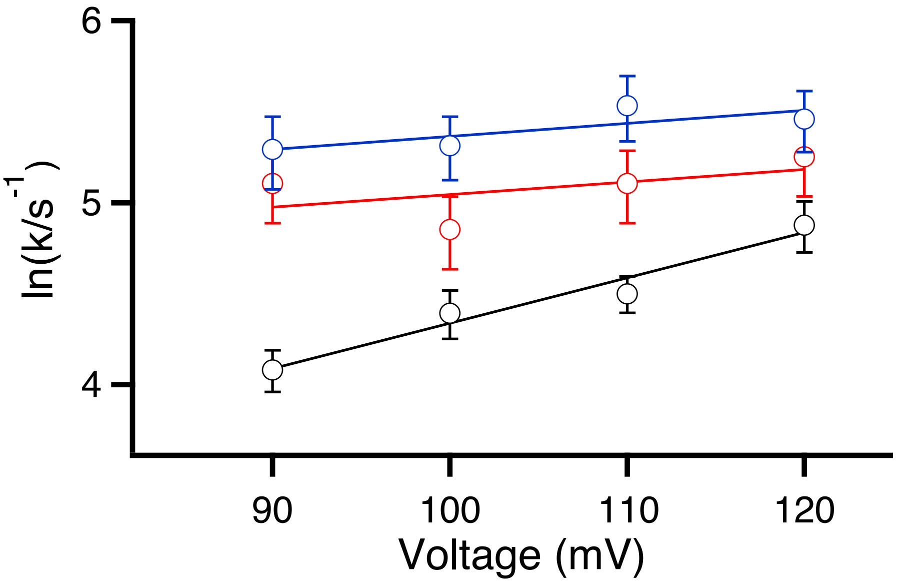

Figure S7. The detachment rates (k_{off}) show a weak dependence on voltage. In black the data for the DNA with the tripartite binding site. In red the data for the DNA with the 1st GAGAG site mutated. In blue the data for the DNA with the 2nd GAGAG site mutated. The lines are the best fit to a straight line. Error bars represent the 95% confidence interval in the rate estimation.
